# Post-Infectious Myocardial Infarction: Does Percutaneous Coronary Intervention Improve Outcomes? A Propensity Score-Matched Analysis †

**DOI:** 10.3390/jcm9061608

**Published:** 2020-05-26

**Authors:** Alain Putot, Frédéric Chagué, Patrick Manckoundia, Philippe Brunel, Jean-Claude Beer, Yves Cottin, Marianne Zeller

**Affiliations:** 1 Geriatrics Internal Medicine Department, University Hospital of Dijon Bourgogne, 21079 Dijon CEDEX, France; patrick.manckoundia@chu-dijon.fr; 2 Physiopathologie et Epidémiologie Cérébro-Cardiovasculaires (PEC2), EA 7460, University of Burgundy and Franche Comté, 21079 Dijon CEDEX, France; frederic.chague@chu-dijon.fr (F.C.); yves.cottin@chu-dijon.fr (Y.C.); marianne.zeller@u-bourgogne.fr (M.Z.); 3 Cardiology Department, University Hospital of Dijon Bourgogne, 21079 Dijon CEDEX, France; jean-claude.beer@chu-dijon.fr; 4 Cardiology Department, Hopital privé Dijon Bourgogne, 21000 Dijon, France; phlppbrunel@gmail.com

**Keywords:** myocardial infarction, type 2 myocardial infarction, acute infection, pneumonia, respiratory tract infection, percutaneous coronary intervention, mortality, outcome, coronary care unit

## Abstract

Acute infection is a frequent trigger of myocardial infarction (MI). However, whether percutaneous coronary intervention (PCI) improves post-infectious MI prognosis is a major but unsolved issue. In this prospective multicenter study from coronary care units, we performed propensity score-matched analysis to compare outcomes in patients with and without PCI for post-infectious MI with angiography-proven significant coronary stenosis (>50%). Among 4573 consecutive MI patients, 476 patients (10%) had a concurrent diagnosis of acute infection at admission, of whom 375 underwent coronary angiography and 321 patients had significant stenosis. Among the 321 patients, 195 underwent PCI. Before the matching procedure, patients without PCI had a similar age and sex ratio but a higher rate of risk factors (hypertension, diabetes, chronic renal failure, and prior coronary artery disease), pneumonia, and SYNTAX score than patients without PCI. After propensity score matching, neither in-hospital mortality (13% with PCI vs. 8% without PCI; *p* = 0.4) nor one-year mortality (24% with PCI vs. 19% without PCI, *p* = 0.5) significantly differed between the two groups. In this first prospective cohort of post-infectious MI in coronary care units, PCI might not improve short- and long-term prognosis in patients with angiography-proven significant coronary stenosis. If confirmed, these results do not argue for systematic invasive procedures after post-infectious MI.

## 1. Introduction

Acute infections, including pneumonia, are increasingly highlighted as triggers of myocardial infarction (MI) [1]. A causal link between infectious triggers and subsequent MI was demonstrated [2]. The mechanisms underlying this association notably include prothrombotic conditions, increased coronary and systemic inflammatory activity, and myocardial metabolic imbalance [2]. Despite the emergence of post-infectious MI as a nosologic entity [2,3] associated with a worsening prognosis, optimal care for post-infectious MI is still unknown. Large retrospective studies from national health insurance data suggest a benefit of revascularization procedures after sepsis-related MI [4,5]. Percutaneous coronary intervention (PCI) is recommended in the emergency care of acute MI due to acute plaque incident (type 1 MI). However, most post-infectious MI cases are type 2 MI [3], characterized by a mismatch between myocardial oxygen supply and demand with no atherothrombotic event, for which such procedures have no proven clinical benefit. Indeed, there is, to date, no evidence on the role of primary PCI in type 2 MI in the literature [6,7]. To our knowledge, only one observational study assessed the impact of revascularization in 491 patients with type 2 MI (T2MI), which failed to highlight any benefit in terms of in-hospital mortality [8]. For T2MI patients with significant stenosis on angiography, the fourth universal definition of MI considers that current guidelines may be applied in accordance with the electrocardiogram (ECG) findings of ST-elevation or non-ST-elevation [9].

In the specific case of post-infectious MI, whether invasive strategies need to be performed remains to be investigated. This lack of evidence leads to a large heterogeneity in management of post-infectious MI, the revascularization (PCI or coronary artery bypass graft surgery (CABG)) rate ranging from 10% to 25% [5,6]. In this first large cohort of post-infectious MI hospitalized in coronary units, we aimed to evaluate the prognostic impact of PCI over the short and long term in patients with significant coronary stenosis (>50%) on coronary angiography.

## 2. Methods

### 2.1. Study Population

The French regional *Observatoire des Infarctus de Côte d’Or* (RICO) survey was described elsewhere [10]. In brief, RICO is an ongoing prospective survey including all consecutive patients hospitalized for MI in all coronary care units (CCU) in one eastern region of France.

**Figure 1:**
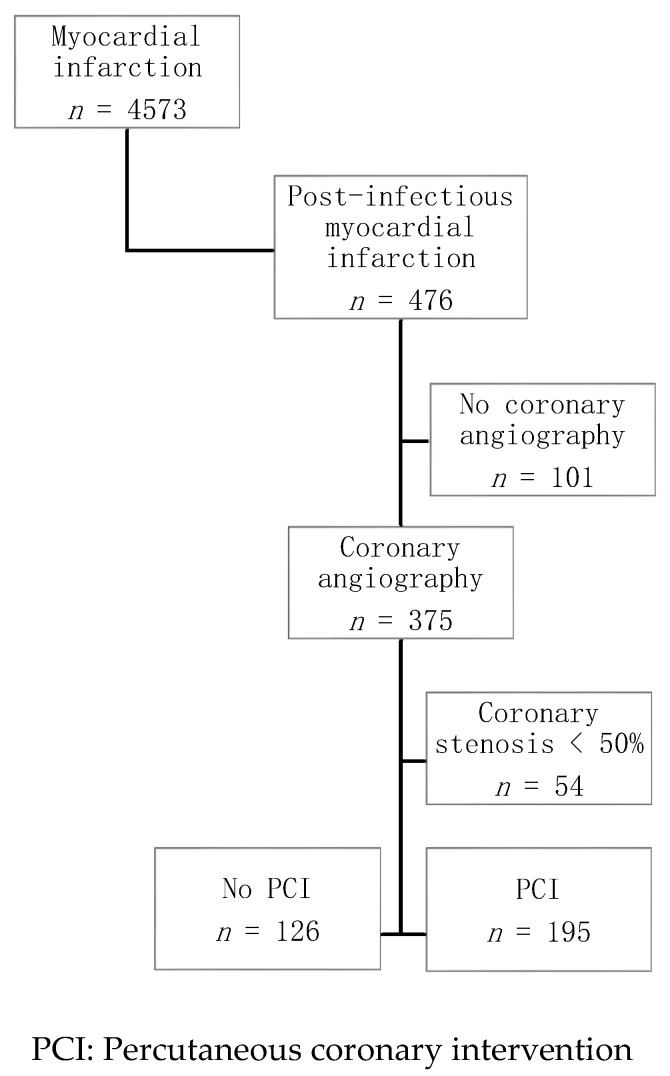
Flow chart.

We included in the present study all consecutive patients admitted from 1 October 2012 to 31 March 2017 for post-infectious MI within 24 h of symptom onset and with angiography-proven coronary stenosis >50% (Figure 1).

The Ethics Committee of the Dijon University Hospital approved the present study. Each patient gave written consent prior to participation.

Given the observational non-randomized design of the study, a propensity score (PS) was built to identify and control for confounding variables that could influence the likelihood of PCI.

### 2.2. Definitions

Post-infectious MI was defined as a concurrent diagnosis of acute infection at the onset of MI symptoms. Acute infection diagnosis was based on the physician’s examination upon admission, by the presence of suggestive signs or symptoms (e.g., cough, sputum, dyspnea, rhonchi, or crackles for respiratory tract infection; dysuria, suprapubic, or flank pain with positive urine culture for urinary tract infection) and at least one of the following criteria of sepsis upon admission: temperature >39 °C, respiratory rate >24 breaths/min, heart rate >100 beats/min, leukocytes >12 × 10^9^/L [11].

Type of MI was defined according to the third universal definition [3]. Type 1 MI was defined on the basis of coronary angiography data, as MI due to a primary coronary event, including plaque erosion or rupture, intraluminal thrombus, or coronary dissection. Type 2 MI was defined by the presence of at least one of the prespecified supply/demand mismatch situations, including acute infection, at the onset of MI symptoms [12], in the absence of documented atherothrombotic event at coronary angiography. According to the universal definition, post-infectious MI, as a situation leading to an acute mismatch between supply and demand in oxygen, was classified as type 2 MI in the absence of evidence of an atherothrombotic event. Type 1/type 2 MI adjudication was investigated by coronary angiography, looking for evidence of acute plaque disruption.

A primary PCI indication was evaluated by the invasive cardiologist on the basis of the clinical, ECG, and angiographic presentation, in accordance with current guidelines [13,14,15]. The benefit/risk balance of an invasive procedure in the frail elderly population was evaluated at the individual level, as recommended [13,16].

Cardiovascular (CV) mortality was defined by the occurrence of a fatal MI, fatal stroke, fatal pulmonary embolism, death due to cardiogenic shock or ventricular rhythm disorders, or sudden unexpected death.

Re-infarction was defined as an acute MI that occurred within 28 days of an incident MI; after 28 days, it was defined as a recurrent MI [9].

### 2.3. Data Collection

Demographic clinical and biological data, as well as ECG, CV risk factors, and history were collected upon admission. The GRACE risk score was also calculated [17]. The following biological parameters were collected: C reactive protein (CRP), hemoglobin level, plasma N-terminal-pro Brain natriuretic peptide (NT-proBNP), creatinine, and estimated glomerular filtration rate (eGFR) using the Chronic Kidney Disease Epidemiology Collaboration formula (CKD-EPI). The troponin Ic peak was obtained from three blood samples taken every 8 h within the first 24 h following admission, using the conventional method (Dimension Vista luminescent oxygen channeling (LOCI™) troponin I assay, Siemens). The left-ventricular ejection fraction (LVEF) was estimated using echocardiography.

Coronary angiography and reperfusion procedures, i.e., PCI and coronary artery bypass graft surgery (CABG), were also recorded. Stenoses ≥50% were considered as significant and <50% diameter stenoses were considered as non-obstructive. The SYNTAX score was determined for each patient [18], as well as hemorrhagic risk, evaluated using the BARC classification [19].

We obtained information on CV events at the one-year follow-up by telephone interview or mail from the patient, patient’s relatives, or treating physician. Only four patients (1%) were lost to follow-up at one year and were excluded from analysis.

### 2.4. Statistical Analyses

Patients with and without PCI were compared using univariate and multivariable analysis. Continuous variables were expressed as medians and interquartile ranges. The Mann–Whitney test was used to compare continuous variables, and the chi-square test was used to compare dichotomous data.

Given the non-randomized design of the study, we used a propensity score (PS) to identify and control for confounding factors that could influence the likelihood of PCI. A multivariable logistic regression model was built to estimate PCI risk and calculate the PS for PCI. The variables included in the multivariable model with a threshold at 5% were age, hypertension, diabetes, atrial fibrillation, type of infection, ST-segment elevation MI (STEMI), type 2 MI, heart rate, CRP, troponin peak, Nt-pro BNP, SYNTAX score, and number of significant lesions. The quality of the model was evaluated using C-statistics (0.81, 95% confidence interval: 0.76–0.87) and Nagelkerke *R^2^* (0.37). The patients with and without PCI were then paired 1:1 on this PS using a caliper width of 10% of the standard deviation of the PS logit.

Kaplan–Meier curves and the log rank test were performed to compare one-year CV mortality of patients with and without PCI.

We used the SPSS 13.0 software package for all analyses.

## 3. Results

### 3.1. Baseline Characteristics

In total, 476 patients were hospitalized in a CCU for post-infectious MI, of whom 375 underwent coronary angiography and 321 (87%) had significant coronary stenosis (median follow-up time: 356 days (337–367)) (Figure 1). Most had respiratory tract infection (*n* = 219, 68%). Among them, 195 (60%) underwent PCI and 126 (39%) did not.

Pre-match and post-match patient characteristics are presented in Table 1. In the pre-match cohort, median age was similar for both groups (74 years in the PCI group vs. 77 years in the group without PCI (*p* = 0.1)). Compared with patients without PCI, patients with PCI had a lower rate of CV risk factors and history of CAD and were, therefore, treated less with CV chronic drugs. In the PCI group, an acute atherothrombotic event (i.e., type 1 MI) was more common (53% vs. 19%, *p* < 0.001), STEMI was also more frequent, and the troponin Ic peak was higher. Coronary lesions at coronary angiography were less severe in the PCI group. Of note, pneumonia, as an infectious trigger, was less frequent in the PCI group. The microbiological features of acute infections leading to MI were presented elsewhere [3]. Acute management is presented in Table 2.

After matching on PS (post-match cohort), as expected, patients with and without PCI had similar characteristics (Table 1 and Table 2).

### 3.2. Outcomes

After matching, in-hospital outcomes comprising re-infarction, atrial fibrillation, bleeding, and heart failure, as well as mortality, were similar for the two groups.

Furthermore, at the one-year follow-up, re-hospitalization for recurrent MI or heart failure, and all-cause and CV mortality did not significantly differ in the two groups (Table 3).

**Figure 2:**
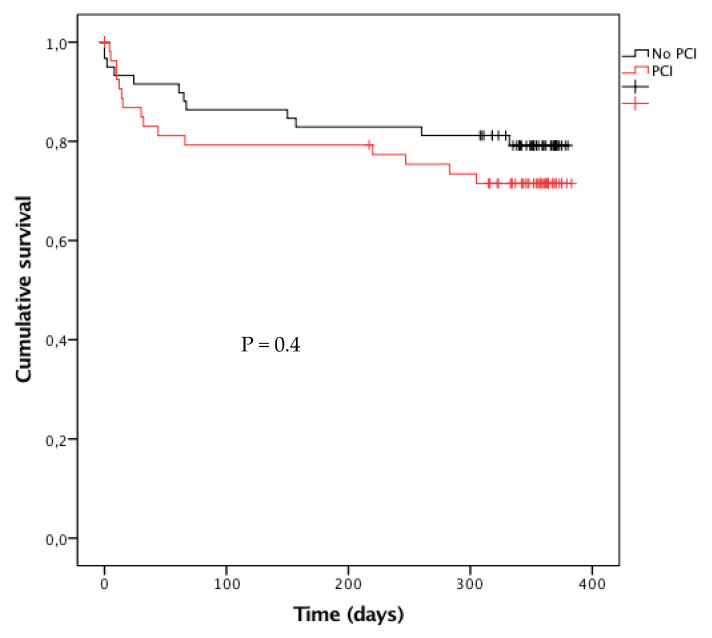
Kaplan–Meier curve of one-year mortality in propensity score-matched population according to percutaneous coronary intervention (PCI) status.

As shown in Figure 2, the Kaplan–Meier curves of one-year mortality in the PS-matched population did not differ in the two groups (log rank: *p* = 0.4).

## 4. Discussion

Acute infection, particularly pneumonia, and MI are both leading causes of hospitalization and mortality and their association, referred to as post-infectious MI, is emerging from the recent literature [1,2,3,20,21]. The present observational study from a multicenter cohort of MI hospitalized in CCU addresses, for the first time, one of the key issues for the acute management of post-infectious MI, namely, PCI, focusing on situations for which such a procedure is relevant, i.e., patients with angiography-proven significant coronary stenosis.

Whether acute invasive strategies should be performed in patients presenting post-infectious MI was not thoroughly addressed. In a large cohort of patients with sepsis from the American National Inpatient Sample database, the rate of post-infectious MI was 4.5%. Among these patients, only 10% underwent an invasive (i.e., PCI and/or CABG) vs. conservative management strategy. In contrast with our results, revascularization was associated with half-reduced in-hospital mortality, after propensity-score matching [5]. Moreover, in the Taiwan Health insurance coding database, among patients with a first MI diagnosis, 7% had concurrent acute infection. Invasive management was also associated with an improved long-term prognosis [6]. However, in these retrospective studies on coding databases, adjustment for potential CV confounders was limited. Thus, access to invasive procedures after MI could indicate a high level of care, introducing a major bias in prognosis evaluation [22]. In our study, only MI patients hospitalized in a CCU were included and prognostic confounders were prospectively collected. Moreover, matching procedures ensured identical coronary artery disease severity in the two groups (PCI and no PCI). Although not significant, CV death showed a trend toward a higher rate in the PCI group. Two main hypotheses can be proposed to explain this finding. Firstly, despite the matching on a comprehensive assessment of CV severity factors and the patients’ comorbidities, we cannot exclude that the PCI decision denoted MI severity, irrespective of all the prognostic factors evaluated. Only a randomized trial could remove this bias. Secondly, our population was aged and multimorbid; PCI remains an invasive procedure with a higher rate of complications in these patients [23]. Most particularly, the prothrombotic effect of sepsis [24] could be associated with an increase in PCI complications [25]. Of note, in a large retrospective study, Higuchi et al. reported that primary PCI was associated with lower in-hospital mortality in patients with type 1 AMI (OR 0.47; 95% CI 0.40–0.55), but not in those with type 2 MI (OR 1.09; 95% CI 0.62–1.94) [8]. These results are consistent with our report, as post-infectious MIs are mainly type 2 MIs. It is noteworthy that the appropriateness of an invasive strategy in type 2 MI is being evaluated in an ongoing randomized control trial [6]. However, since type 2 MI has heterogeneous causes, therapeutic strategies should also be investigated using a phenotype-specific approach [26].

Several limitations have to be acknowledged regarding these results. Firstly, this study was limited to CCU patients, although post-infectious MI frequently occurs in frail comorbid individuals hospitalized in other non-CCU departments [22]. This inclusion bias could lead to an overestimation of type 1 post-infectious MI, given that type 2 MI is less frequent in cardiology departments. However, since type 2 MI patients are less likely to benefit from PCI [27,28], this limitation does not argue for invasive procedures. Secondly, the distinction between type 2 MI and myocardial injury diagnosis is a key issue, because the latter is commonly associated with acute infection [9]. However, only patients with MI diagnosed on the basis of clinical, imaging, or ECG signs were included in this study. Thirdly, although this procedure is not 100% sensitive for plaque disruption, type 1/type 2 MI differentiation was made based on angiographic data. Intracoronary imaging with optical coherence tomography or intravascular ultrasound can be helpful to highlight a plaque incident [29]; however, they were not available in this study. Fourthly, diagnosis of acute infections in the setting of an acute MI is often unclear, since MI itself is responsible for an intense inflammatory response [30], and clinical distinction between acute respiratory infection and heart failure is sometimes difficult. Fifthly, a contemporary troponin assay was used, whereas small-scale myocardial injury patients might have been missed. Finally, the sample size was markedly reduced after PS matching. As patients undergoing PCI largely differ from those without PCI, matching was only applicable for half of the PCI patients. Therefore, no firm conclusions could be drawn from this small observational study, and randomized trials are, therefore, urgently needed.

## 5. Conclusions

In a small prospective cohort study of CCU patients hospitalized for post-infectious MI with angiography-proven significant coronary stenosis, one-fifth of the patients died at one year, mostly from CV causes, and PCI was not associated with an improved prognosis. Due to the small sample size, these results can be seen as hypothesis-generating and as a call for data from randomized controlled trials. If confirmed, these results do not argue for systematic invasive procedures at the acute phase of post-infectious MI.

## Figures and Tables

**Table 1:** Patient characteristics at admission (*n* (%) or median (interquartile range)).

	PCI *n* = 195	No PCI *n* = 126	*p*	PCI (Post-Match) *n* = 62	No PCI (Post-Match) *n* = 62	*p*
**Risk factors and comorbidities**						
Age, years	74 (61–83)	77 (67–83)	0.1	76 (65–83)	77 (69–83)	0.5
Female	80 (41)	40 (32)	0.09	34 (50)	29 (48)	0.4
Hypertension	122 (63)	99 (79)	0.002	46 (74)	48 (77)	0.7
Hypercholesterolemia	96 (49)	66 (52)	0.6	30 (48)	30 (48)	1
Family history of CAD	50 (26)	27 (21)	0.4	17 (27)	12 (19)	0.3
Smoking	100 (51)	75 (59)	0.1	28 (45)	33 (53)	0.4
Diabetes	67 (34)	59 (47)	0.03	27 (44)	29 (47)	0.7
Chronic renal failure	20 (10)	24 (19)	0.02	7 (11)	9 (14)	0.6
COPD	33 (17)	26 (21)	0.4	10 (16)	9 (14)	0.8
Neoplasia	33 (17)	21 (17)	1	10 (16)	9 (14)	0.8
**Cardiovascular history**						
CAD	57 (29)	56 (44)	0.005	13 (21)	20 (32)	0.2
Stroke	20 (10)	17 (13)	0.4	10 (16)	6 (10)	0.3
PAD	26 (13)	29 (23)	0.02	12 (19)	8 (13)	0.3
HF	13 (7)	13 (10)	0.2	4 (6)	2 (3)	0.4
Atrial fibrillation	10 (5)	20 (16)	0.001	4 (6)	5 (8)	0.7
Aortic stenosis	14 (7)	14 (11)	0.2	4 (6)	7 (11)	0.3
**Type of MI**						
Type 1	103 (53)	24 (19)	<0.001	22 (35)	16 (26)	0.2
Type 2	92 (47)	102 (81)	<0.001	40 (63)	46 (71)	0.2
**Type of infection**						
Bronchitis	96 (49)	45 (36)	0.02	23 (44)	24 (36)	0.9
Pneumonia	37 (19)	41 (33)	0.006	19 (24)	16 (26)	0.5
Urinary tract infection	28 (14)	22 (17)	0.5	11 (18)	11 (18)	1
Other infections	34 (17)	18 (14)	0.5	8 (13)	8 (13)	1
**Clinical data**						
HR, beats/min	80 (67–96)	85 (73–103)	0.004	81 (70–100)	81 (72–94)	1
SBP, mmHg	135 (114–156)	135 (118–153)	0.8	130 (117–155)	136 (119–161)	0.5
DBP, mmHg	76 (65–90)	75 (62–82)	0.1	72 (64–88)	75 (62–83)	0.7
GRACE risk score	168 (139–192)	177 (153–197)	0.1	172 (141–203)	171 (151–189)	0.7
Acute HF *	92 (47)	72 (57)	0.08	37 (60)	36 (58)	0.9
LVEF < 40%	66 (35)	48 (39)	0.4	22 (36)	18 (30)	0.5
Cardiac arrest	14 (7)	3 (2)	0.06	3 (5)	1 (2)	0.6
**ECG**						
STEMI	100 (51)	40 (32)	0.001	29 (47)	23 (37)	0.3
AF/flutter	17 (9)	21 (17)	0.09	8 (13)	6 (10)	0.6
LBBB	21 (11)	14 (11)	0.4	5 (8)	8 (13)	0.4
**Biological data**						
Hemoglobin, g/100 mL	13.2 (12.2–15.0)	12.4 (10.6–13.6)	0.001	13.1 (11.6–15.4)	13.7 (10.5–14.1)	0.1
Leukocytes, G/L	13.6 (12.1–14.8)	11.1 (9.5–15.1)	0.2	13.9 (12.7–19.5)	11.2(6.7–14.2)	0.1
CRP, mg/L	12 (4–78)	49 (10–105)	<0.001	35 (6–122)	24 (5–89)	0.7
eGFR, mL/min	69 (48–89)	57 (37–84)	0.01	69 (46–88)	58 (39–78)	0.1
Troponin I peak, µg/L	17 (4–72)	6 (1–20)	<0.001	13 (4–53)	10 (1–23)	0.1
NT-proBNP, pg/mL	1950 (368–6302)	4475 (1673–10915)	<0.001	4187 (968–18634)	4491 (1565–9626)	0.7
**Angiographic data**						
1-Vessel disease	68 (35)	29 (23)	0.02	20 (32)	17 (27)	0.6
2-Vessel disease	57 (29)	31 (25)	0.4	19 (31)	18 (29)	0.8
3-Vessel disease or Left main	70 (36)	66 (52)	0.004	23 (37)	27 (43)	0.5
SYNTAX score	11 (6–19)	19 (11–28)	<0.001	14 (8–20)	15 (10–21)	0.5
**Chronic treatment**						
Aspirin	60 (31)	50 (40)	0.1	19 (31)	19 (31)	1
Other antiplatelets	33 (17)	35 (28)	0.02	11 (18)	15 (24)	0.4
Beta-blockers	75 (38)	59 (47)	0.1	21 (34)	24 (39)	0.6
CCB	46 (24)	39 (31)	0.1	17 (24)	22 (35)	0.8
ARB	40 (20)	36 (29)	0.1	13 (21)	21 (34)	0.1
ACEI	45 (23)	46 (37)	0.009	18 (29)	17 (27)	0.8
Statins	72 (37)	66 (52)	0.006	19 (31)	28 (45)	0.1
Diuretics	79 (40)	63 (50)	0.09	29 (47)	32 (52)	0.6
AVK/DOA	22 (11)	23 (18)	0.08	8 (13)	12 (19)	0.3

* Killip class > 1; ACEI: angiotensin-converting enzyme inhibitors; ARB: angiotensin receptor blockers; AVK: anti-vitamin K; AF: atrial fibrillation; CABG: coronary artery bypass grafting; CAD: coronary artery disease; CCB: calcium channel blockers; CK: creatine kinase; COPD: chronic obstructive pulmonary disease; CRP: C-reactive protein; DBP: diastolic blood pressure; DOA: direct oral anticoagulant; eGFR: estimated glomerular filtration rate; HF: heart failure; HR: heart rate; LBBB: left-bundle branch block; LVEF: left-ventricular ejection fraction; MI: myocardial infarction; NT-proBNP: N-terminal-pro brain natriuretic peptide; PAD: peripheral arterial disease; PCI: percutaneous coronary intervention; SBP: systolic blood pressure; STEMI: ST-segment elevation myocardial infarction.

**Table 2:** Acute management (*n* (%)).

	PCI *n* = 195	No PCI *n* = 126	*p*	PCI (Post-Match) *n* = 62	No PCI (Post-Match) *n* = 62	*p*
**Reperfusion procedures**						
Thrombolysis	5 (3)	1 (1)	0.4	1 (2)	0	1
CABG	3 (2)	14 (11)	0.07	2 (3)	7 (1)	0.2
**First 48-h treatment**						
Amines	26 (14)	10 (8)	0.1	8 (13)	3 (5)	0.2
Aspirin	183 (97)	115 (94)	0.2	60 (97)	59 (95)	1
Other antiplatelets	179 (95)	99 (81)	<0.001	59 (95)	51 (82)	0.04
Beta-blockers	123 (65)	80 (65)	1	37 (60)	43 (69)	0.3
CCB	49 (26)	31 (25)	0.9	16 (26)	21 (34)	0.3
ARB	13 (7)	11 (9)	0.5	2 (3)	6 (10)	0.3
ACEI	83 (44)	46 (37)	0.3	30 (48)	16 (26)	0.009
Statins	158 (84)	111 (90)	0.1	51 (82)	59 (95)	0.04
Diuretics	88 (47)	76 (62)	0.008	33 (53)	40 (64)	0.2
Nitrates	84 (44)	41 (33)	0.05	34 (55)	23 (37)	0.05
LMWH	130 (67)	69 (56)	0.02	41 (66)	33 (53)	0.1
UFH	56 (30)	50 (41)	0.05	22 (35)	24 (39)	0.7
AVK/DOA	19 (10)	17 (13)	0.3	5 (8)	10 (16)	0.3
**Treatment at discharge**						
Aspirin	168 (89)	104 (85)	0.3	53 (85)	53 (85)	1
Other antiplatelets	162 (86)	45 (37)	<0.001	47 (76)	19 (31)	<0.001
Beta-blockers	138 (73)	94 (76)	0.5	41 (66)	48 (77)	0.2
CCB	30 (16)	22 (18)	0.6	14 (23)	11 (18)	0.5
ARB	13 (7)	13 (11)	0.2	2 (3)	7 (11)	0.2
ACEI	132 (70)	61 (50)	<0.001	47 (76)	27 (43)	<0.001
Statins	157 (83)	99 (81)	0.6	46 (74)	50 (81)	0.4
Diuretics	67 (35)	67 (54)	0.001	24 (39)	37 (60)	0.02
Nitrates	20 (11)	18 (15)	0.3	11 (18)	5 (8)	0.1
AVK/DOA	27 (14)	38 (30)	<0.001	9 (15)	18 (29)	0.01

ACEI: angiotensin-converting enzyme inhibitors; ARB: angiotensin receptor blockers; AVK: anti-vitamin K; CABG: coronary artery bypass grafting; CCB: calcium channel blockers; DOA: direct oral anticoagulant; LMWH: low-molecular-weight heparin; PCI: percutaneous coronary intervention, UFH: unfractionated heparin.

**Table 3:** Outcomes (*n* (%)).

	PCI *n* = 195	No PCI *n* = 126	OR (95% CI)	*p*	PCI (Post-Match) *n* = 62	No PCI (Post-Match) *n* = 62	OR (95% CI)	*p*
**In-hospital events**								
ICU stay >5 days	69 (35)	54 (43)	0.73 (0.46–1.15)	0.2	34 (55)	32 (52)	0.88 (0.43–1.78)	0.7
Severe HF *	56 (29)	41 (32)	0.83 (0.51–1.36)	0.5	21 (34)	19 (31)	1.16 (0.55–2.46)	0.7
VT/VF	15 (8)	6 (5)	1.66 (0.59–5.39)	0.3	6 (10)	3 (5)	2.09 (0.42–13.6)	0.5
Atrial fibrillation	22 (12)	23 (19)	1.74 (0.88–3.47)	0.2	8 (13)	13 (21)	0.56 (0.18–1.61)	0.3
Bleeding #	14 (7)	22 (17)	0.37 (0.17–0.79)	0.004	7 (11)	11 (18)	0.59 (0.18–1.82)	0.3
Re-infarction	8 (4)	4 (3)	1.31 (0.39–4.43)	0.7	4 (6)	3 (5)	1.36 (0.29–6.33)	0.7
All-cause mortality	17 (9)	12 (9)	0.91 (0.42–1.97)	0.8	8 (13)	5 (8)	1.69 (0.52–5.48)	0.4
CV mortality	15 (8)	11 (9)	0.87 (0.39–1.96)	0.7	7 (11)	5 (8)	1.45 (0.43–4.85)	0.5
**One-year events**								
Recurrent MI	9 (5)	5 (5)	1.12 (0.52–2.20)	0.8	4 (6)	2 (3)	2.00 (0.34–11.0)	0.5
Hospitalization for HF	7 (4)	8 (6)	0.52 (0.18–1.48)	0.2	2 (3)	6 (10)	0.29 (0.06–1.51)	0.2
All-cause mortality	35 (18)	30 (24)	0.71 (0.41–1.22)	0.2	15 (24)	12 (19)	1.33 (0.56–3.13)	0.5
CV mortality	29 (15)	16 (13)	1.17 (0.61–2.27)	0.6	13 (21)	8 (13)	1.73 (0.66–4.54)	0.3

* Killip Class 3–4, # BARC 3 or 5 bleeding; CI: confidence interval; CV: cardiovascular; HF: heart failure; ICU: intensive care unit; MI: myocardial infarction; PCI: percutaneous coronary intervention; OR: odds ratio; VT/VF: ventricular tachycardia/ventricular fibrillation.

## References

[1] Musher D.M., Abers M.S., Corrales-Medina V.F. (2019). Acute Infection and Myocardial Infarction. N. Engl. J. Med..

[2] Corrales-Medina V.F., Madjid M., Musher D.M. (2010). Role of acute infection in triggering acute coronary syndromes. Lancet Infect. Dis..

[3] Putot A., Chague F., Manckoundia P., Cottin Y., Zeller M. (2019). Post-Infectious Myocardial Infarction: New Insights for Improved Screening. J. Clin. Med..

[4] Smilowitz N.R., Gupta N., Guo Y., Bangalore S. (2016). Comparison of Outcomes of Patients with Sepsis With Versus Without Acute Myocardial Infarction and Comparison of Invasive Versus Noninvasive Management of the Patients With Infarction. Am. J. Cardiol..

[5] Liu E.-S., Chiang C.-H., Hung W.-T., Tang P.-L., Hung C.C., Kuo S.-H., Liu C.-P., Chen Y.-S., Mar G.-Y., Huang W.-C. (2019). Comparison of long-term mortality in patients with acute myocardial infarction associated with or without sepsis. Int. J. Infect. Dis..

[6] Lambrakis K., French J.K., Scott I.A., Briffa T., Brieger D., Farkouh M.E., White H., Chuang A.M.-Y., Tiver K., Quinn S. (2019). The appropriateness of coronary investigation in myocardial injury and type 2 myocardial infarction (ACT-2): A randomized trial design. Am. Heart J..

[7] DeFilippis A.P., Chapman A.R., Mills N.L., de Lemos J.A., Arbab-Zadeh A., Newby L.K., Morrow D.A. (2019). Assessment and Treatment of Patients with Type 2 Myocardial Infarction and Acute Nonischemic Myocardial Injury. Circulation.

[8] Higuchi S., Suzuki M., Horiuchi Y., Tanaka H., Saji M., Yoshino H., Nagao K., Yamamoto T., Takayama M. (2019). Higher non-cardiac mortality and lesser impact of early revascularization in patients with type 2 compared to type 1 acute myocardial infarction: Results from the Tokyo CCU Network registry. Heart Vessels.

[9] Thygesen K., Alpert J.S., Jaffe A.S., Chaitman B.R., Bax J.J., Morrow D.A., White H.D., Thygesen K., Alpert J.S., ESC Scientific Document Group (2019). Fourth universal definition of myocardial infarction (2018). Eur. Heart J..

[10] Zeller M. (2004). Impaired fasting glucose and cardiogenic shock in patients with acute myocardial infarction. Eur. Heart J..

[11] Levy M.M., Fink M.P., Marshall J.C., Abraham E., Angus D., Cook D., Cohen J., Opal S.M., Vincent J.-L., Ramsay G. (2003). 2001 SCCM/ESICM/ACCP/ATS/SIS International Sepsis Definitions Conference. Crit. Care Med..

[12] Putot A., Jeanmichel M., Chague F., Manckoundia P., Cottin Y., Zeller M. (2020). Type 2 Myocardial Infarction: A Geriatric Population-based Model of Pathogenesis. Aging Dis.

[13] Amsterdam E.A., Wenger N.K., Brindis R.G., Casey D.E., Ganiats T.G., Holmes D.R., Jaffe A.S., Jneid H., Kelly R.F., Kontos M.C. (2014). 2014 AHA/ACC Guideline for the Management of Patients with Non-ST-Elevation Acute Coronary Syndromes: A report of the American College of Cardiology/American Heart Association Task Force on Practice Guidelines. J. Am. Coll. Cardiol..

[14] Bassand J.-P., Hamm C.W., Ardissino D., Boersma E., Budaj A., Fernández-Avilés F., Fox K.A.A., Hasdai D., Ohman E.M., Task Force for Diagnosis and Treatment of Non-ST-Segment Elevation Acute Coronary Syndromes of European Society of Cardiology (2007). Guidelines for the diagnosis and treatment of non-ST-segment elevation acute coronary syndromes. Eur. Heart J..

[15] Ibanez B., James S., Agewall S., Antunes M.J., Bucciarelli-Ducci C., Bueno H., Caforio A.L.P., Crea F., Goudevenos J.A., Halvorsen S. (2018). 2017 ESC Guidelines for the management of acute myocardial infarction in patients presenting with ST-segment elevation: The Task Force for the management of acute myocardial infarction in patients presenting with ST-segment elevation of the European Society of Cardiology (ESC). Eur. Heart J..

[16] Rich M.W., Chyun D.A., Skolnick A.H., Alexander K.P., Forman D.E., Kitzman D.W., Maurer M.S., McClurken J.B., Resnick B.M., Shen W.K. (2016). Knowledge Gaps in Cardiovascular Care of the Older Adult Population: A Scientific Statement from the American Heart Association, American College of Cardiology, and American Geriatrics Society. Circulation.

[17] Granger C.B., Goldberg R.J., Dabbous O., Pieper K.S., Eagle K.A., Cannon C.P., Van De Werf F., Avezum A., Goodman S.G., Flather M.D. (2003). Predictors of hospital mortality in the global registry of acute coronary events. Arch. Intern. Med..

[18] Sianos G., Morel M.-A., Kappetein A.P., Morice M.-C., Colombo A., Dawkins K., van den Brand M., Van Dyck N., Russell M.E., Mohr F.W. (2005). The SYNTAX Score: An angiographic tool grading the complexity of coronary artery disease. EuroIntervention.

[19] Mehran R., Rao S.V., Bhatt D.L., Gibson C.M., Caixeta A., Eikelboom J., Kaul S., Wiviott S.D., Menon V., Nikolsky E. (2011). Standardized bleeding definitions for cardiovascular clinical trials: A consensus report from the Bleeding Academic Research Consortium. Circulation.

[20] Kwong J.C., Schwartz K.L., Campitelli M.A., Chung H., Crowcroft N.S., Karnauchow T., Katz K., Ko D.T., McGeer A.J., McNally D. (2018). Acute Myocardial Infarction after Laboratory-Confirmed Influenza Infection. New Engl. J. Med..

[21] Meier C.R., Jick S.S., Derby L.E., Vasilakis C., Jick H. (1998). Acute respiratory-tract infections and risk of first-time acute myocardial infarction. Lancet.

[22] D’Souza M., Saaby L., Poulsen T.S., Diederichsen A.C.P., Hosbond S., Diederichsen S.Z., Larsen T.B., Schmidt H., Gerke O., Hallas J. (2014). Comparison of mortality in patients with acute myocardial infarction accidentally admitted to non-cardiology departments versus that in patients admitted to coronary care units. Am. J. Cardiol..

[23] Feldman D.N., Gade C.L., Slotwiner A.J., Parikh M., Bergman G., Wong S.C., Minutello R.M. (2006). New York State Angioplasty Registry Comparison of outcomes of percutaneous coronary interventions in patients of three age groups (<60, 60 to 80, and >80 years) (from the New York State Angioplasty Registry). Am. J. Cardiol..

[24] Modica A., Karlsson F., Mooe T. (2007). Platelet aggregation and aspirin non-responsiveness increase when an acute coronary syndrome is complicated by an infection. J. Thromb. Haemost..

[25] Piccaro de Oliveira P., Gonzales V., Lopes R.D., Schmidt M.M., Garofallo S., Santos R.P.D., Carrion L., Gottschall C., Quadros A.S. (2016). Serious infections among unselected patients with ST-elevation myocardial infarction treated with contemporary primary percutaneous coronary intervention. Am. Heart J..

[26] Januzzi J.L., Sandoval Y. (2017). The Many Faces of Type 2 Myocardial Infarction. J. Am. Coll. Cardiol..

[27] Putot A., Derrida S.B., Zeller M., Avondo A., Ray P., Manckoundia P., Cottin Y. (2018). Short-Term Prognosis of Myocardial Injury, Type 1 and Type 2 Myocardial Infarction in the Emergency Unit. Am. J. Med..

[28] Neumann J.T., Sörensen N.A., Rübsamen N., Ojeda F., Renné T., Qaderi V., Teltrop E., Kramer S., Quantius L., Zeller T. (2017). Discrimination of patients with type 2 myocardial infarction. Eur. Heart J..

[29] Sandoval Y., Jaffe A.S. (2019). Type 2 Myocardial Infarction. J. Am. Coll. Cardiol..

[30] Frangogiannis N.G. (2014). The inflammatory response in myocardial injury, repair, and remodelling. Nat. Rev. Cardiol..

